# Incidence of Genome Structure, DNA Asymmetry, and Cell Physiology on T-DNA Integration in Chromosomes of the Phytopathogenic Fungus *Leptosphaeria maculans*

**DOI:** 10.1534/g3.112.002048

**Published:** 2012-08-01

**Authors:** Salim Bourras, Michel Meyer, Jonathan Grandaubert, Nicolas Lapalu, Isabelle Fudal, Juliette Linglin, Benedicte Ollivier, Françoise Blaise, Marie-Hélène Balesdent, Thierry Rouxel

**Affiliations:** * Institut National de la Recherche Agronomique (INRA), Research Unit 1290 BIOGER, F-78850 Thiverval-Grignon, France, and; † INRA, Research Unit 1290 BIOGER, F-78026 Versailles Cedex, France

**Keywords:** T-DNA, mutagenesis, *Leptosphaeria maculans*, genome structure, Gene Ontology

## Abstract

The ever-increasing generation of sequence data is accompanied by unsatisfactory functional annotation, and complex genomes, such as those of plants and filamentous fungi, show a large number of genes with no predicted or known function. For functional annotation of unknown or hypothetical genes, the production of collections of mutants using *Agrobacterium tumefaciens*–mediated transformation (ATMT) associated with genotyping and phenotyping has gained wide acceptance. ATMT is also widely used to identify pathogenicity determinants in pathogenic fungi. A systematic analysis of T-DNA borders was performed in an ATMT-mutagenized collection of the phytopathogenic fungus *Leptosphaeria maculans* to evaluate the features of T-DNA integration in its particular transposable element-rich compartmentalized genome. A total of 318 T-DNA tags were recovered and analyzed for biases in chromosome and genic compartments, existence of CG/AT skews at the insertion site, and occurrence of microhomologies between the T-DNA left border (LB) and the target sequence. Functional annotation of targeted genes was done using the Gene Ontology annotation. The T-DNA integration mainly targeted gene-rich, transcriptionally active regions, and it favored biological processes consistent with the physiological status of a germinating spore. T-DNA integration was strongly biased toward regulatory regions, and mainly promoters. Consistent with the T-DNA intranuclear-targeting model, the density of T-DNA insertion correlated with CG skew near the transcription initiation site. The existence of microhomologies between promoter sequences and the T-DNA LB flanking sequence was also consistent with T-DNA integration to host DNA mediated by homologous recombination based on the microhomology-mediated end-joining pathway.

The first eukaryotic (and fungal genome) to be sequenced was that of the budding yeast *Saccharomyces cerevisiae* (Goffeau *et al.* 1996). Since then, an ever-expanding number of fungal genomes has been made available, and the genome sequence of more than 300 isolates from more than 150 fungal species is currently available or in progress (http://cfgp.riceblast.snu.ac.kr/main.php; http://fungalgenomes.org/wiki/Fungal_Genome_Links), with prospects for more fungal genome sequencing, such as the 1000 fungal genome initiative (Grigoriev *et al.* 2011). Whereas high-throughput approaches, such as transcriptomics, proteomics, and comparative genomics between related species, have proved useful in eukaryotic genome annotation to predict the correct gene structure, functional annotation lags behind, and complex genomes, such as those of plants and filamentous fungi, show a large number of genes with no predicted or known function [*e.g.*, Arabidopsis Genome Initiative (2000)]. The dramatic increase in whole-genome sequencing is thus accompanied by a dramatic difficulty to reach the full biological value of the sequenced genomes with accurate identification of the protein-coding genes, as well as the nature of the functional protein products. In yeast and in some model plants, such as *Arabidopsis thaliana*, this was partly achieved with the involvement of a wide community, which promoted the development of strain/line collections in which virtually every protein-coding gene in the genome was modified, for example, by deleting, tagging with green fluorescent protein (GFP), or engineering for overexpression (Jones *et al.* 2008; Huh *et al.* 2003; Winzeler *et al.* 1999; Alonso *et al.* 2003). Even with this wide involvement of the research community, *ca*. 1000 of 5796 (17%) of protein-encoding genes in yeast and *ca*. one third of *A. thaliana* proteins still lack a functional annotation to date (Pena-Castillo and Hughes 2007; Kourmpetis *et al.* 2011).

Although the production of large collections of mutants with disrupted or inactivated genes associated with genotyping and phenotyping has gained wide acceptance for functional annotation of unknown or hypothetical genes, this has only been developed in a few tractable model plant or fungal species, mainly using *Agrobacterium tumefaciens*–mediated transformation (ATMT) (Alonso *et al.* 2003; Michielse *et al.* 2005; Krishnan *et al.* 2009; Thole *et al.* 2010). Furthermore, the whole-genome investigation for T-DNA tag distribution and the biases linked with integration conditioning the possibility to reach saturation mutagenesis has only been investigated for very few model plant species [*i.e.*, *A. thaliana*, rice, and *Brachypodium distachyon* (Alonso *et al.* 2003; Krishnan *et al.* 2009; Thole *et al.* 2010)], and only one phytopathogenic filamentous fungus, *Magnaporthe oryzae* (Choi *et al.* 2007; Meng *et al.* 2007).

Filamentous fungi, including *M. oryzae*, were first believed to have compact genomes with very few repeated elements and repeat-rich genomic regions. Filamentous fungi are amenable to ATMT (Michielse *et al.* 2005). Furthermore, biases linked with T-DNA integration in the genome of *M. oryzae* were indicated to be lower than in plants and the T-DNA integration was suggested to be “more canonical” than in plants (Choi *et al.* 2007). However, the sequencing of numerous fungal species indicates an extreme diversity of genomic complexity, genome size, and genomic landscapes, ranging from those fungi with compact genomes to fungi where massive transposable element (TE) invasion took place, eventually resulting in genome sizes larger than that of *A. thaliana* (*e.g.*, Spanu *et al.* 2010). Filamentous fungi with complex genomes also are characterized by compartmentalized, “two-speed” genomes in which specific compartments of the genome, usually TE-rich, are also enriched in genes involved in niche adaptation, such as pathogenicity effectors in phytopathogenic fungi. Examples of this encompass dispensable ‘B’ chromosomes of *Fusarium* (Ma *et al.* 2010), TE-rich regions of the powdery mildew fungi (Spanu *et al.* 2010), and AT-rich isochores comprising one third of the genome of *Leptosphaeria maculans* (Rouxel *et al.* 2011). By comparison with *M. oryzae*, there is only preliminary information on how T-DNA integration will happen in such genomes and how it will be useable for saturation mutagenesis of genes involved in niche adaptation. For example, in the case of the fungal pathogen of oilseed rape, *L. maculans*, analysis of 135 T-DNA integration events at a time when the genome sequence was not available indicated T-DNA preferentially integrated as a single copy in gene-rich regions of the fungal genome, but not in AT isochores (Blaise *et al.* 2007). The low frequency of T-DNA tags corresponding to known or putative protein-coding genes (19.3%) also suggested a probable bias toward intergenic and/or regulatory regions (Blaise *et al.* 2007). However, as underlined by some authors (Meng *et al.* 2007), the absence of genome sequence for *L. maculans* limited the analyses that could be performed (*i.e.*, on favored targets for T-DNA integration), and conclusions about the possible bias toward promoter regions drawn by Blaise *et al.* (2007) could not be substantiated.

The objective of this article was to further evaluate the suitability of ATMT for random saturation mutagenesis in the compartmentalized fungal genome of *L. maculans* and to further establish the mechanism of T-DNA integration in fungal genomes, taking advantage of an increased collection of T-DNA–mutagenized isolates and availability of the *L. maculans* genome sequence. One of the main questions to be addressed regarded the accessibility of AT isochores of the genome and thus of genes involved in pathogenicity by the T-DNA. A total of 400 T-DNA tags were generated, and their pattern of integration in the genome was investigated in terms of chromosomal biases, distribution within chromosomes, distribution within protein-coding regions, and targeted motifs. In addition, a Gene Ontology (GO) annotation was done and compared with that of the whole genome to identify possible insertion biases due to the physiological stage of the germinating conidia at the time of ATMT process.

## Materials and Methods

### Transformation of *L. maculans* germinating conidia

All *L. maculans* transformants were issued from the *A. tumefaciens*–mediated transformation (ATMT) of the reference isolate v23.1.3 (Attard *et al.* 2002) sequenced by Genoscope (Rouxel *et al.* 2011). Generation of the collection was described by Blaise *et al.* (2007) and increased for this study to *ca*. 5000 T-DNA–tagged lines. Briefly, ATMT was performed on germinating conidia using the *A. tumefaciens* strain C58pGV2260 harboring the binary vector pBBH. The vector contains a hygromycin B resistance gene (*hph*) under the control of the *Aspergillus nidulans* gpdA promoter (Blaise *et al.* 2007).

### Definition of genome compartment for insertion of T-DNA tags

*L. maculans* chromosomes were first compartmentalized based on nucleic acids composition in AT-rich and GC-equilibrated isochores as described in Rouxel *et al.* (2011).

Following the automated annotation of the genome (Rouxel *et al.* 2011) and regardless of the isochore structure of the genome, we generated four gene-centered collections of sequences: (i) coding regions, defined as sequences from start to stop codons, and further subdivided to introns and exons; (ii) gene-promoter regions; (iii) terminator regions; and (iv) intergenic regions, defined as the remaining genomic sequences. Regulatory regions, and mainly promoters, are often ill-defined in fungi. For this reason, when analyzing T-DNA tag position relative to genes in the *M. oryzae* genome, Meng *et al.* (2007) defined three sets of 500 bp, 750 bp, and 1000 bp for 5′ promoter regions and downstream regulatory regions. However, the increase in size of regulatory regions did not drastically change the features associated with the T-DNA–favored target (Meng *et al.* 2007). In addition, the genome of *L. maculans* is more compact than that of *M. oryzae* in GC isochores [*e.g.*, median size of intergenic regions in the case of head-to-tail ORFs is 670 bp (Rouxel *et al.* 2011)]. We thus only investigated here one range of size for promoters and terminators: 500 bp upstream of gene start codons or downstream of gene stop codons, respectively. Collections of gene-promoter, terminator, and intergenic regions were extracted using a Python script, departing from gene coordinates.

### Recovery of T-DNA–flanking sequences and analysis of T-DNA–targeted genes

T-DNA–flanking sequences were recovered from genomic DNA by thermal asymmetric interlaced-PCR (tail-PCR) and PCR-walking techniques as described in Liu *et al.* (1995) and Balzergue *et al.* (2001), respectively. Sequencing was performed on PCR products using a Beckman Coulter CEQ 8000 automated sequencer (Beckman Coulter, Fullerton, CA, USA) according to the manufacturer's instructions. All sequences were cured manually and aligned to *L. maculans* genome sequence using BLASTn with a cutoff e-value of 1e−10. The latter step was automated using a homemade script in Python. The position of an insertion site was defined as the position of the first aligned nucleotide to a flanking sequence. All extracted positions were mapped and plotted on the *L. maculans*–assembled genome using homemade scripts in Python and R. Based on mapping of T-DNA insertion sites, genes with a T-DNA tag in their promoter, terminator, or coding region were extracted, mapped, and analyzed for size, compositional, and structural features. The latter step was automated using homemade scripts in Python.

### Functional annotation using GO

GO annotations of *L. maculans*–predicted genes were done with Blast2GO (Götz *et al.* 2008) as described in Rouxel *et al.* (2011). The NCBI “NR” database (October 16, 2009, release version) was queried with all predicted genes using BLAST algorithm version 2.2.21 on the URGI high-throughput computing cluster (128 Intel Xeon E5450). All genes were mapped according to GO, GeneInfo, Gene2accession, and PIR data, and then analyzed with Blast2GO, which applies GO annotations from BLAST search results. This process takes into account sequence similarity and the evidence code (EC) associated with GO annotations. Finally, GO annotations were enriched using Annex and Interproscan data. In this work, we chose to use the “biological process” vocabulary for functional annotation and comparison between T-DNA–tagged genes and all genes of the genome, because this GO vocabulary was found to better fit fungal behavior when described from a physiological or phenotypical point of view. In addition, it is the vocabulary for which the highest annotation number was obtained in yeast (Christie *et al.* 2009).

### Statistical analyses

Biases were assessed by calculating the standardized residuals between observed and expected values as follows: SR = (Observed – Expected) / √Expected. SR calculation allows the detection of outlying observations [*i.e.*, those that appear to deviate from other members of the sample in which they occur (Grubbs 1969)]. In general, SR > 0 means the observed value is greater than expected, and by contrast, SR < 0 means the observed value is smaller than expected. To test whether the outlying observations deviate significantly from what is expected, the SR distribution following a normal distribution was estimated using the Kolmogorov-Smirnov test embedded in XLSTAT statistical analysis software version 2009.6.02 (with default parameters). Therefore, when the hypothesis of normal distribution was not rejected, SR exceeding the absolute value of 1.96 was considered a bias (*i.e.*, significantly deviant from the rest of the data).

The Monte Carlo test on contingency tables was used as an alternative to assess biases of T-DNA tags mapping. This nonparametric test based on simulations assesses the independence between rows and columns. Then, when coupled with Fisher's exact test, it determines whether the difference between the observed and the theoretical values is significant. All calculations were performed using the appropriate XLSTAT function with default parameters.

The linear regression option of XLSTAT was used to model the relationships between data sets. A graphical output comprising the regression line and the 95% confidence intervals area was generated using the embedded function of the software.

## RESULTS

### Generation of the repertoire of T-DNA–flanking sequences

A subset of the collection of 5000 transformants of *L. maculans* obtained by ATMT was selected for sequencing the T-DNA insertion borders. Four-hundred sequences were obtained. Of these, 40 T-DNA–flanking sequences were generated by PCR walking (Balzergue *et al.* 2001) and 360 by tail-PCR (Liu *et al.* 1995). BLASTn searches against the *L. maculans* genome indicated 33 sequences (8.25%) had no BLAST hit (sequences too short for the BLASTn algorithm and sequences corresponding to the bacterial vector). The remaining 367 sequences were filtered for ambiguous BLAST hits (poor homology below the cutoff e-value of 1e−10), resulting in the final repertoire of 318 flanking sequences corresponding to single-locus T-DNA integration events in unique transformants. Of these, 217 sequences were obtained by sequencing the left border (LB) of the T-DNA insertion, and 101 by sequencing its right border (RB).

### Compartmentalization of the genome and T-DNA integrations

**Figure 1:**
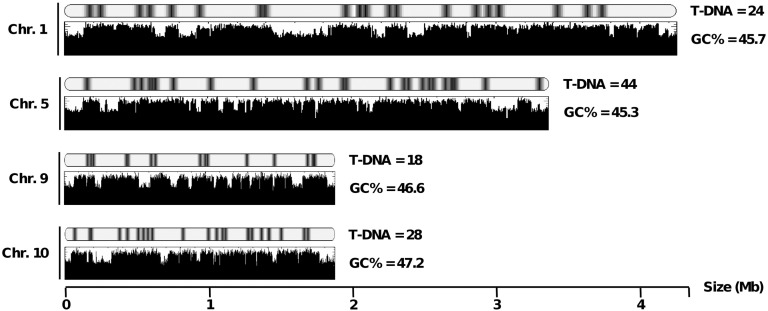
A schematic representation of occurrence of T-DNA insertion events along four *L. maculans* chromosomes. For each chromosome, the upper plot shows the location of the T-DNA integration events, and the lower plot schematizes variations in GC content along the chromosome, defining AT-rich and GC-equilibrated isochores. The average GC percentage of the chromosome is indicated.

The *L. maculans* genome is compartmentalized into two distinct genomic landscapes: GC isochores (summing up 64% of the genome and containing 95% of the genes) and AT isochores (summing up 36% of the genome and 5% of the genes, but mainly consisting of mosaics of inactivated and truncated TEs) (Rouxel *et al.* 2011). The T-DNA insertions were graphically coincident to GC isochores in an almost systematic fashion (Figure 1), and 96.9% of T-DNA tags were mapped to GC isochores *vs.* only 3.1% that were mapped to AT isochores. AT isochores are further subdivided into three categories: telomeres (representing *ca*. 2% of the genome assembly); large-sized isochores (13–325 kb, representing *ca*. 31% of the genome assembly) corresponding to complex mosaics of TEs; and mid-sized isochores (1–13 kb, summing up *ca*. 3% of the genome assembly) generally corresponding to the insertion of a single TE within a GC isochore (Rouxel *et al.* 2011). The above-mentioned depletion of T-DNA integration in AT isochores was mainly due to a very low amount of integrations in large AT isochores with only two cases observed (0.6%), whereas 1.6% of the tags were found in telomeres that may contain active genes, including numerous copies of a RecQ telomere-linked helicase (Rouxel *et al.* 2011). No T-DNA tag was coincident with multiple loci in the genome, even in AT-rich compartments and telomeres, due to sequence divergence generated by RIP acting on repeated copies of TEs. Actually, even when mapping to a given TE family, the tag sequence, when unambiguous, was always sufficient to derive a single locus due to these sequence polymorphisms.

In addition to these two distinct compartments, the rDNA array summing up 1.7% of the genome assembly (Rouxel *et al.* 2011) was also underrepresented with no T-DNA tags targeting it.

### Chromosomal features and T-DNA integrations

**Figure 2:**
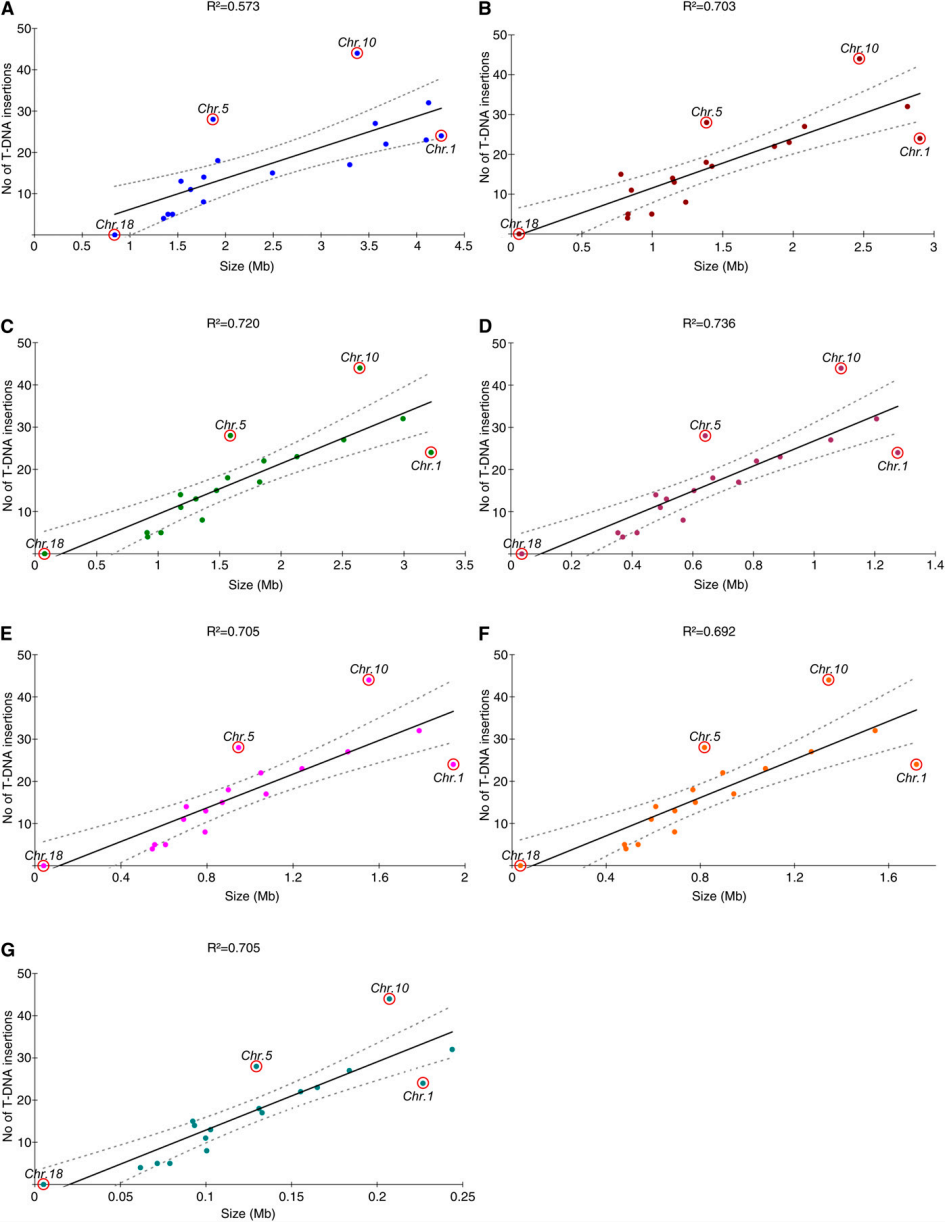
Correlation between the number of T-DNA integrations and chromosomal features. The features investigated for each chromosome were (A) chromosome size; (B) total size of the GC isochores; (C) total size of the transcriptional regions [defined as the sum of regulatory sequences (promoter + terminator) and gene-coding sequences (exons + introns)]; (D) total size of the regulatory regions (defined as the sum of promoter and terminator sequences); (E) total size of gene-coding regions (defined as the sum of exonic and intronic sequences); (F) total size of the exonic sequences; and (G) total size of the intronic sequences. Regression curves and the 95% confidence intervals are plotted in continuous and discontinuous lines, respectively.

The number of T-DNA insertions per chromosome was then compared with seven chromosomal features (Figure 2), and the distribution of T-DNA insertions was plotted against each of these features. Globally, the number of T-DNA integrations was linearly correlated with all investigated features, but it better correlated with size of the GC isochores within chromosomes (*R^2^* = 0.703) than with total size of the chromosome (*R^2^* = 0.573). The favored insertion sites were transcriptional regions (*R^2^* = 0.720), mainly regulatory regions (*R^2^* = 0.736) and introns (*R^2^* = 0.705) (Figure 2). Most chromosomes, except chromosomes 5, 10, and to a lesser extent, 1, showed such a linear correlation between the number of T-DNA integrations and chromosomal features (see below).

### Favored T-DNA insertion events in genic regions

Noticing that the chromosomal distribution of T-DNA tags was correlated to the size of gene-regulatory and gene-coding regions within chromosomes, we studied to what extent compartmentalization features are involved in profiling whole-genome T-DNA insertion occurrence. In contrast to what is described in *M. oryzae* (Meng *et al.* 2007; Choi *et al.* 2007), targeting of ORF was not significantly different in *L. maculans* to what would be expected under the hypothesis of random integration in the genome (Table 1). Biases assessment using the SR method showed that T-DNA insertions were less common than expected in intergenic regions (SR = −6.67) and exons (SR = −2.04) and more common in gene regulatory regions (SR = 12.92) and gene introns (SR = 4.24) (Table 1). Biases in favor of regulatory regions were corroborated by the Monte-Carlo test. However, no significant bias was observed in intergenic regions and introns according to this analysis (data not shown).

**Table 1:** Distribution of T-DNA insertion events within *L. maculans* genomic regions

Genomic Regions				T-DNA Insertion Events		
Type	Size (Mb)	% Genome		Observed	Expected*^a^*	SR*^b^*
Regulatory*^c^*	11.8	26		200	83	12.92
5′ promoting*^c^*	5.9	13		122	41	12.64
3′ terminating*^c^*	5.9	13		78	41	5.77
Coding*^c^*	17.6	39		119	123	−0.37
Exons	15.3	34		86	107	−2.04
Introns	2.3	5		33	16	4.24
Shared*^c^*	—	—		41	—	
Intergenic*^c^*	15.7	35		40	110	−6.67

a Expected number of T-DNA integration events (T-IE) [= (T-IE genomic density) × (genomic region size)]. Values were approximated to the nearest integer.

b Standardized residues. We considered a normal distribution of SRs because we cannot reject the null hypothesis as revealed by the Kolmogorov-Smirnov test (*P*-value = 0.976, α = 0.05).

c Regulatory regions, defined as the sum of promoting and terminating regions of the 12,469 predicted genes of *L. maculans*; Gene-promoting regions, 500 bp upstream of the start codon; Gene-terminating regions, 500 bp downstream of the stop codon; Gene-coding regions, from start to stop codons, including introns; Shared, common regulatory regions shared by two head-to-tail nearby genes; Intergenic, genomic regions corresponding to none of the previous criteria. Note that overlaps between compartments may occur, leading to a total number of sequences higher than 318.

### Promoter features favoring T-DNA targeting

**Figure 3:**
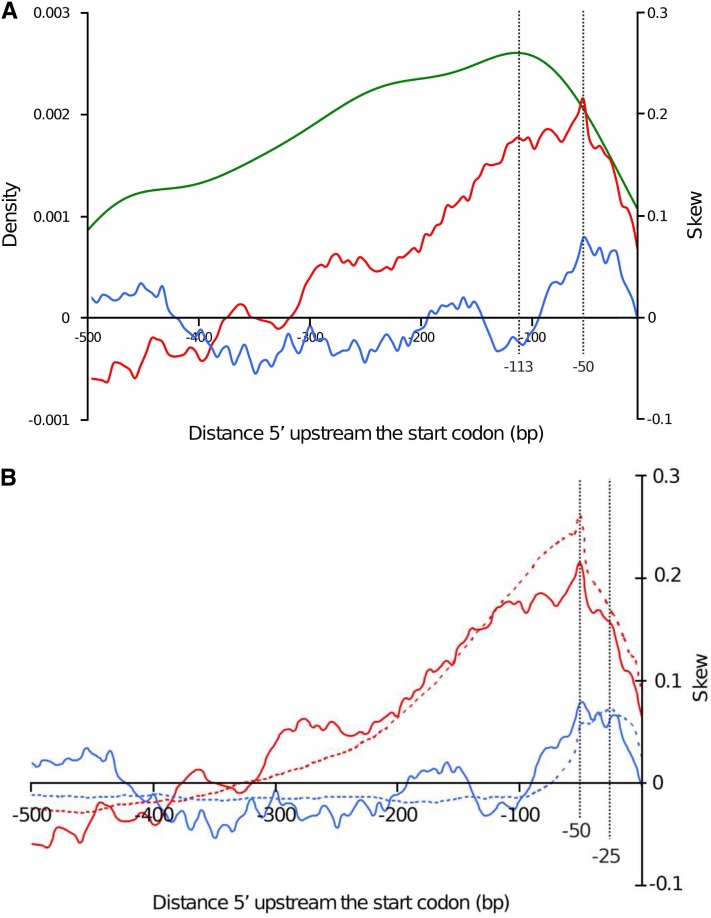
The link between CG skew and AT skew in gene promoter regions and favored T-DNA integration events. A. Density of T-DNA insertions in promoter regions (green curve), CG skew (red curve) and AT skew (blue curve) variations along T-DNA-targeted gene promoter regions, as a function of location from the ATG. B. Comparison of CG skew (red curve) and AT skew (blue curve) variations between promoter regions of T-DNA–targeted genes (plain lines) and promoters of all *L. maculans* predicted genes (dotted lines).

Because promoters are the main genomic regions in which T-DNA integration occurred, we analyzed further promoter regions to investigate the involvement of host-DNA asymmetry and T-DNA–host-DNA shared microhomologies to favor the T-DNA targeting. Previous studies noticed an increased CG skew around transcription start site in *A. thaliana* and other eukaryotes (Tatarinova *et al.* 2003). The targeted promoter sequences were thus analyzed for DNA asymmetry by calculating CG and AT skews. Positive CG and AT skew values indicate an overabundance of C and A residues, respectively, whereas negative CG and AT skew values indicate an overabundance of G and T residues, respectively. Sequences 500 bp upstream the transcription initiation start codon of 122 T-DNA–targeted genes harboring a T-DNA tag in their promoter region were first extracted, and then CG and AT skews were calculated, plotted, and compared with the density of T-DNA integration events in the same intervals (Figure 3A). The T-DNA tag density and CG skew increased gradually when getting closer from the start codon, to reach their maximum value at positions −113 and −50 respectively, and then decreased dramatically (Figure 3A), whereas CG and AT skews plotted differently but shared the same peak position at −50. To corroborate the functional meaning of CG skew peak in promoters targeted by T-DNA, we compared it with the CG skew profile of whole *L. maculans* promoters following the extraction of sequences 500 bp upstream of the transcription initiation start codon for all 12,469 *L. maculans*–predicted genes. Comparison of AT/CG skews between promoters of both collections showed that, in both cases, CG skews reached their peak value at position −50. By contrast, AT skew peak in whole-genome promoters profile plotted at position −25, closer to gene start (Figure 3B).

To assess to what extent DNA asymmetry impacted T-DNA integration, the occurrence of CG and AT skews contexts at T-DNA insertion sites were also analyzed in gene terminator, gene coding and intergenic regions. Sequences 200 bp upstream and downstream the 318 insertion sites were extracted and split out into three groups: (i) gene terminator regions (78 sequences), (ii) gene coding regions (119 sequences) and (iii) intergenic regions (40 sequences). For comparison purposes, the 122 sequences corresponding to gene promoter regions were added.

**Figure 4:**
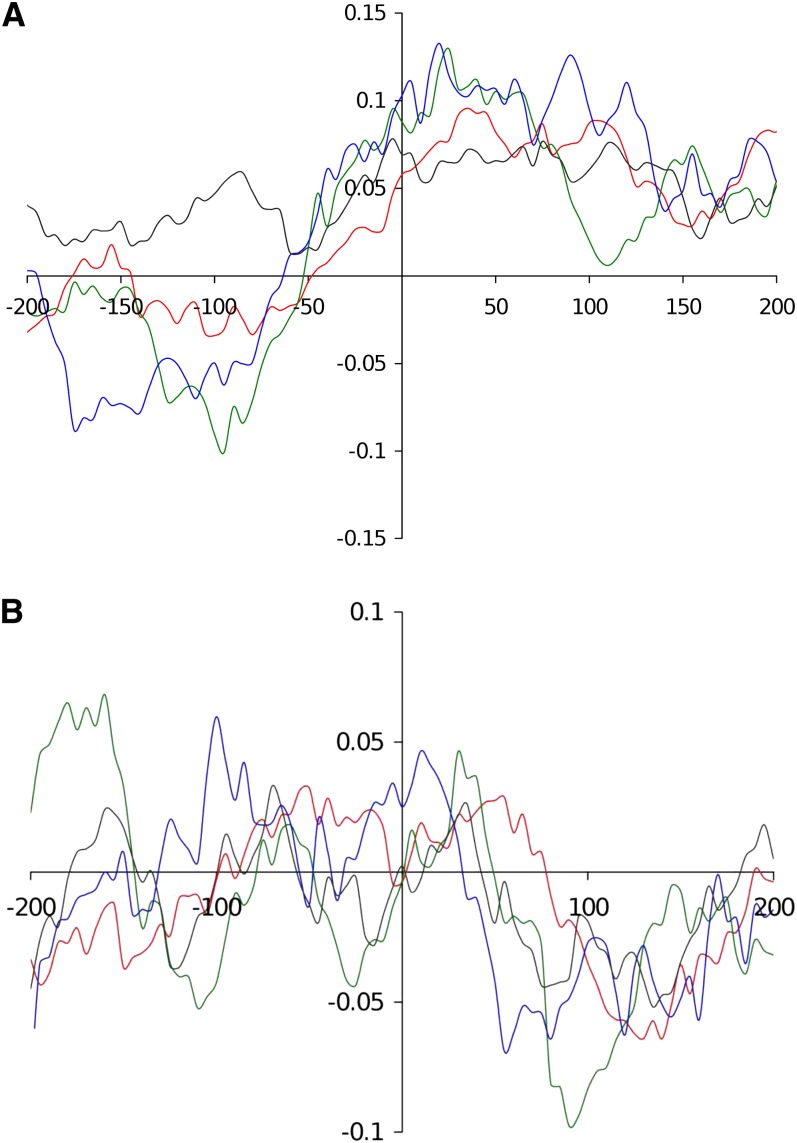
Analysis of CG (A) and AT skews (B) at T-DNA insertion sites in four targeted compartments of the genome. Sequences 200 bp upstream and downstream of the integration sites were extracted and CG/AT skews were calculated. The sequences were then grouped according to four compartments of the genome: promoter (red curves), terminator (green curves), intergenic (blue curves), and protein coding (black curves) regions.

Skew graphics showed that T-DNA insertions occurred preferentially in increased CG skew context, in all genomic compartments (Figure 4A) and also in a weak AT skewed context for promoter, terminator and gene coding regions. In contrast, AT skew was increased at the insertion sites in intergenic regions (Figure 4B).

### Microhomologies between the T-DNA left border and T-DNA preinsertion sites

T-DNA integration to host DNA is mediated by two major mechanisms: nonhomologous recombination (NHR) and homologous recombination (HR) via the T-DNA LB [for review, see Tzfira *et al.* (2004) and Citovsky *et al.* (2007)]. The former is in fact a HR-like mechanism relying on the microhomology-mediated end-joining (MMEJ) pathway and its property to use 5–25 bp microhomologous sequences to anneal and join free single-stranded DNA ends [for review, see McVey and Lee (2008)]. We thus investigated whether microhomologies between the 25-bp T-DNA LB and host DNA could be found at the insertion site.

**Figure 5:**
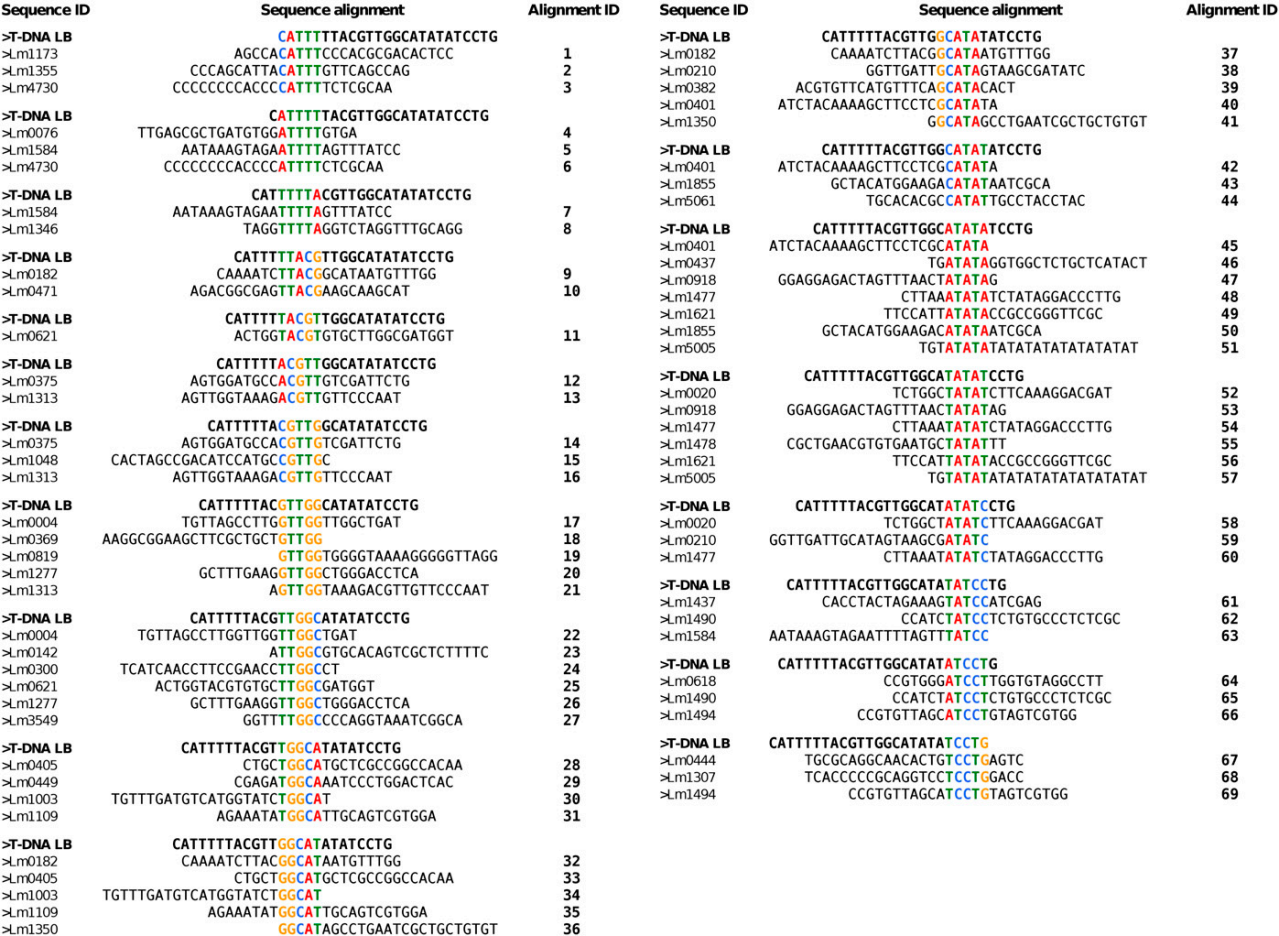
The search for microhomology between the host-DNA and T-DNA left border. One hundred and sixty 25-bp preinsertion sites were investigated for occurrence of 5-bp-long consecutive motifs corresponding to identical motifs in the T-DNA left border. The 41 sequences of preinsertion sites that show identity with consecutive, 1-bp sliding window, and 5-bp-long motifs are displayed.

Sequences 25-bp backward of the insertion sites were extracted and chosen so that (i) they correspond to sequences upstream of a junction between T-DNA LB and host DNA; (ii) they were exempt from potential filler DNA at T-DNA–host-DNA junction; and (iii) they were exempt from gaps and undetermined nucleotides (N) in the current version of genome assembly (Rouxel *et al.* 2011). A total of 160 25-bp sequences was thus obtained. We also divided the T-DNA LB into 5-bp-long successive sequences that we named “microhomology motifs” and aligned the 21 resulting motifs to the 160 selected preinsertion sites (Figure 5). Nineteen putative microhomology motifs were found in 69 locations, distributed unequally among 41 preinsertion sites (25.6%) (Figure 5). No single motif was common to all sequences, but 3 microhomology motifs (14.2%) TTGGC (Figure 5, alignments 22–27), ATATA (Figure 5, alignments 45–53), and TATAT (Figure 5, alignments 54–62), were found in 32.4% of the locations, suggesting the presence of homology islands. In addition, TATA-containing motifs were the most represented (32.4%) (Figure 5, alignments 42–65).

**Figure 6:**
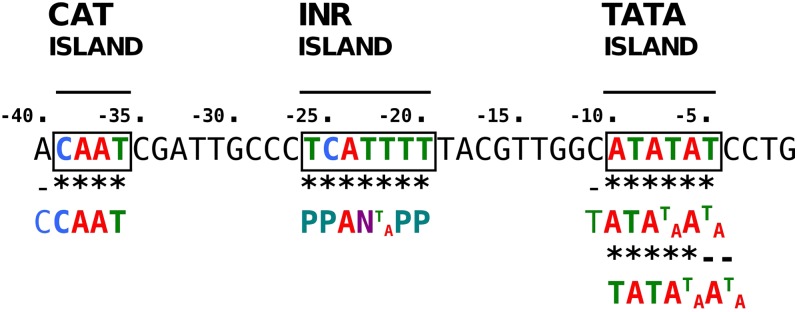
Occurrence of sequence microhomologies to eukaryotic core promoter elements (TATA box, CAT box, and Initiator) in the T-DNA LB and 15 upstream supplementary bases.

**Figure 7:**
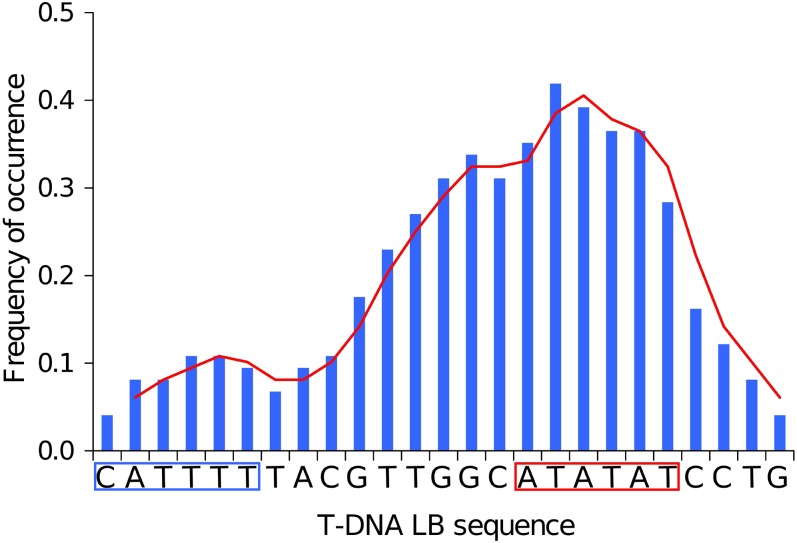
Analysis of microhomology at T-DNA preinsertion sites. Frequency of occurrence of single bases identical to those of the 25-bp T-DNA left border in the genome preinsertion sites were analyzed. The T-DNA LB sequence is illustrated, and homologs of the TATA box and Inr in the LB sequence are boxed.

Assessment of T-DNA LB sequence affinity with common core promoter elements of eukaryotic genes (TATA box with consensus TATA(T/A)A(A/T) (Breathnach and Chambon 1981; Burley 1996), CAT box with core consensus CCAAT (Bucher 1990), and initiator (Inr) with consensus PPAN(T/A)PP (P, pyrimidine; N, any nucleotide) (Javahery *et al.* 1994) showed that the 25-bp-long T-DNA LB harbored islands of homologies with both TATA box (positions −5 to −10) and Inr (positions −20 to −25) elements. In addition, when we extended the alignment to 15 upstream supplementary bases, an additional homology with CAT box was found (Figure 6). We lastly calculated the frequency of occurrence of each base of the T-DNA LB sequence in microhomology motifs and plotted the values along the T-DNA LB sequence. As shown in Figure 7, T-DNA–host-DNA base identity increased approaching the LB free end. Corroborating the previous observation, the TATA island, but not Inr island, can be postulated to be frequently represented in T-DNA–host-DNA shared microhomologies due to the high ratio of identical bases at this location (Figure 7).

### GO annotation of T-DNA–targeted genes

The whole-genome mapping of the 318 T-DNA insertions showed that 279 of these were in gene-coding or regulatory regions, whereas the other T-DNA tags were located in intergenic regions, including AT-rich, gene-poor isochores. A functional profile of the collection of T-DNA–targeted genes was performed by coupling the GO annotation of the “biological process” vocabulary with an assessment of representation bias by calculating the SRs between observed and expected number of annotations per functional category. The proportion of genes coding for hypothetical or predicted proteins of unknown function in the T-DNA–targeted gene collection (73.1%) was comparable to that observed in the whole genome (71.8%). Most of the functional categories represented in the “biological process” vocabulary (15 of 22) were represented in genes tagged by T-DNA (Table 2). The SR values for “pigmentation” (SR = 5.94), “growth” (SR = 2.09), and “cell wall organization or biogenesis” (SR = 3.21) revealed an overrepresentation in the T-DNA–targeted gene collection compared with all predicted genes of the genome, whereas the “signaling” functional category was underrepresented (SR = −2.03) (Table 2). Similar biases were identified using the Monte Carlo method (data not shown).

**Table 2:** Gene Ontology annotation of T-DNA–targeted genes using the “biological process” vocabulary

Whole Genome		T-DNA–targeted Genes		
	Annot.*^a^*	Obs. Annot.*^b^*	Exp. Annot.*^c^*	SR*^d^*
**Pigmentation**	**1**	**1**	**0.03**	**5.94**
Immune system process	1	0	0.03	−0.16
Cell proliferation	3	0	0.08	−0.28
Death	4	0	0.11	−0.33
Locomotion	4	0	0.11	−0.33
Biological adhesion	6	0	0.16	−0.40
**Growth**	**6**	**1**	**0.16**	**2.09**
Nitrogen utilization	9	0	0.24	−0.49
Reproduction	14	1	0.38	1.02
Carbon utilization	15	1	0.40	0.94
Multi-organism process	43	1	1.15	−0.14
**Cell wall organization or biogenesis**	**49**	**5**	**1.31**	**3.21**
**Signaling**	**153**	**0**	**4.11**	**−2.03**
Cellular component organization	177	5	4.75	0.11
Multicellular organismal process	228	6	6.12	−0.05
Cellular component biogenesis	232	8	6.23	0.71
Developmental process	257	8	6.90	0.42
Response to stimulus	262	4	7.03	−1.14
Biological regulation	469	12	12.59	−0.17
Localization	706	21	18.95	0.47
Cellular process	2489	62	66.79	−0.59
Metabolic process	3070	84	82.39	0.18
Total	8198	220	220	—

a Number of annotations generated per category for the 12,469 *L. maculans* predicted genes.

b Observed number of annotations generated by the GO analysis.

c Expected number of annotations [= (∑annot.) × P(functional category)]. Where (∑annot.) is the sum of all generated annotations, and P(functional category) is whole genome probability of the considered functional category. Values were approximated to two decimals.

d Standardized residuals. We considered a normal distribution of SRs because we cannot reject the null hypothesis as revealed by the Kolmogorov-Smirnov test (*P*-value = 0.391, α = 0.05). Biased categories are indicated in bold.

### Functional significance of chromosome bias in T-DNA insertions?

T-DNA insertion events were mapped onto the *L. maculans* genome and plotted along its 18 chromosomes to investigate distribution biases. The T-DNA insertion density varied from 0 insertion event/Mb (chromosome 18) to 14.7 insertion events/Mb (chromosome 11) (Table 3). In most of the cases, the number of tags per chromosome was compliant with a random integration of the T-DNA. However, T-DNA insertion events were found to be statistically more common than expected into chromosomes 5 (SR = 4.08) and 10 (SR = 4.16) (Table 3 and Figure 2), whereas they were less common than expected into chromosome 18 (SR = −2.45) (Table 3). Chromosome 1, whose number of T-DNA integrations was consistent with chromosome size (Figure 2), showed a number of tags markedly lower than the mean confidence intervals for other criteria, such as size of coding or regulatory regions (Figure 2).

**Table 3:** Distribution of T-DNA insertion events along the *L. maculans* chromosomes

Chromosomes							T-DNA Insertion Events			
No.	SC*^a^*	Size (Mb)	GC %	Gene Content*^b^*	GC Size*^c^* (Mb)		ρ*^d^* (T-IE/Mb)	Observed	Expected*^e^*	SR*^f^*
1	0	4.3	45.7	1276	2.9		5.6	24	30	−1.06
2	2+19	4.1	44.1	1206	2.8		7.8	32	29	0.58
3	6+29+11	3.7	39.7	810	1.9		6.0	22	26	−0.74
4	8+10	3.6	43.2	1055	2.1		7.6	27	25	0.41
5	1	**3.4**	**45.3**	**1089**	**2.5**		**13.0**	**44**	**24**	**4.19**
6	12+15+32	4.1	41.6	888	2.0		5.6	23	29	−1.06
7	20+21+23	3.3	44.7	751	1.4		5.2	17	23	−1.27
8	3+31	2.5	43.3	604	0.8		6.0	15	17	−0.58
9	4	1.9	46.6	666	1.4		9.4	18	13	1.25
10	5	**1.9**	**47.2**	**641**	**1.4**		**15.0**	**28**	**13**	**4.12**
11	9	1.8	45.1	478	1.1		7.9	14	12	0.45
12	7	1.8	46.0	568	1.2		4.5	8	12	−1.25
13	13	1.6	43.9	493	0.9		6.7	11	11	−0.13
14	14	1.5	47.4	513	1.2		8.5	13	11	0.69
15	17	1.4	43.7	416	1.0		3.5	5	10	−1.61
16	16	1.4	44.3	353	0.8		3.6	5	10	−1.53
17	18	1.4	44.7	369	0.8		3.0	4	9	−1.77
18	22	**0.8**	**35.3**	**36**	**0.1**		**0.0**	**0**	**6**	**-2.42**
Un.*^g^*	—	0.7	—	—	—		—	8	—	—
Genome	—	45.1	44.1	12469	—		7.0	318		

a Supercontigs reassembled to make up whole chromosomes.

b Number of predicted genes per chromosome.

c Total size of GC isochores per chromosome.

d T-DNA insertion event (T-IE) density [= (number of T-IEs per chromosome / chromosome size)].

e Based on density of T-DNA insertion events in the whole genome (7 T-IE/Mb), the expected number of T-IEs per chromosome was calculated as [(chromosome size) × (T-IE whole-genome density)]. Values were approximated to the nearest integer.

f Standardized residuals. We considered a normal distribution of SRs because we cannot reject the null hypothesis as revealed by the Kolmogorov-Smirnov test (*P*-value = 0.475, α = 0.05). Chromosomes showing a significant bias in number of T-DNA insertion events are indicated in bold.

g Unassembled genomic sequences (summing up approximately to 0.7 Mb).

Chromosome 18 is very rich in TE and poor in genes (Rouxel *et al.* 2011), and the lack of T-DNA integration in this chromosome is consistent with the above-mentioned preferred integration in gene-rich genomics regions. GO annotation indicated that chromosome 5 was significantly enriched in the “pigmentation” (SR = 2.93) and “carbon utilization” (SR = 2.14) functional categories, whereas chromosome 10 showed no significant enrichment in any functional categories (Table 4). Of these, only the “pigmentation” functional category was found to be overrepresented in the collection of T-DNA–targeted genes (Table 2). These data suggest that functional bias is unrelated to genome distribution of T-DNA insertions. Biases assessment using the Monte Carlo method led to the same results (data not shown).

**Table 4:** Gene Ontology annotation of “biological process” for chromosomes 5 and 10

Whole Genome		Chromosome 5			Chromosome 10		
	Annot.*^a^*	Obs. Annot.*^b^*	Exp. Annot.*^c^*	SR*^d^*	Obs. Annot.*^b^*	Exp. Annot.*^c^*	SR*^d^*
**Pigmentation**	**1**	**1**	**0.10**	**2.93**	**0**	**0.06**	**-0.24**
Immune system process	1	0	0.10	−0.31	0	0.06	−0.24
Cell proliferation	3	0	0.29	−0.54	0	0.17	−0.41
Death	4	1	0.38	1.00	0	0.22	−0.47
Locomotion	4	1	0.38	1.00	0	0.22	−0.47
Biological adhesion	6	1	0.57	0.56	0	0.33	−0.58
Growth	6	1	0.57	0.56	1	0.33	1.15
Nitrogen utilization	9	1	0.86	0.15	0	0.50	−0.71
Reproduction	14	2	1.34	0.57	2	0.78	1.38
**Carbon utilization**	**15**	**4**	**1.43**	**2.14**	**1**	**0.83**	**0.18**
Multi-organism process	43	4	4.11	−0.05	0	2.39	−1.55
Cell wall organization or biogenesis	49	5	4.68	0.15	2	2.73	−0.44
Signaling	153	14	14.61	−0.16	7	8.51	−0.52
Cellular component organization	177	19	16.91	0.51	11	9.85	0.37
Multicellular organismal process	228	19	21.78	−0.59	11	12.68	−0.47
Cellular component biogenesis	232	31	22.16	1.88	17	12.90	1.14
Developmental process	257	21	24.55	−0.72	12	14.30	−0.61
Response to stimulus	262	16	25.02	−1.80	22	14.57	1.95
Biological regulation	469	47	44.79	0.33	31	26.09	0.96
Localization	706	62	67.43	−0.66	33	39.27	−1.00
Cellular process	2489	240	237.73	0.15	149	138.45	0.90
Metabolic process	3070	293	293.22	−0.01	157	170.76	−1.05
Total	8198	783	783	—	456	456	_

a Number of annotations generated per category for the 12,469 genes predicted in *L. maculans*.

b Observed number of annotations generated for all predicted genes on chromosome 5 and 10.

c Expected number of annotations [= (∑annot.) x P(functional category)]. Where (∑annot.) is the sum of all generated annotations, and P(functional category) is whole genome probability of the considered functional category. Values were approximated to two decimals.

d Standardized residuals. We considered a normal distribution of SRs because we cannot reject the null hypothesis as revealed by the Kolmogorov-Smirnov test (Chromosome 5: *P*-value = 0.453, α = 0.05. Chromosome 10: *P*-value = 0.188, α = 0.05). Biased categories are indicated in bold.

## DISCUSSION

Although many fungal species are amenable to ATMT, the mechanisms of T-DNA integration in the fungal genomes are largely unknown compared with what is known in plants, and it is still a matter of debate to know whether T-DNA integration will be random enough to allow a systematic targeting of all genes in the genome for functional identification. In phytopathogenic filamentous fungi, numerous pathogenicity mutants were generated by ATMT, but a systematic analysis of T-DNA integration in the genomes has only been performed in *M. oryzae* (Choi *et al.* 2007; Meng *et al.* 2007) and in *L. maculans* prior to obtainment of the whole-genome sequence (Blaise *et al.* 2007). Here we exploited the *L. maculans* genome sequence to investigate how “canonical” T-DNA integration patterns are in a fungal genome with such contrasted genomic landscapes compared with what is known in *M. oryzae*. This comparison, however, has to be taken with care, as the two articles on *M. oryzae* show some experimental differences with ours. In Choi *et al.* (2007), a very large number of 1246 transformants were investigated, but more than 1100 were chosen so that they harbor phenotypic defects, thus suggesting a bias toward T-DNA integration within coding sequences, in detriment to lines in which noncoding regions were targeted. In this sense, Meng *et al.* (2007), who reported on characterization of a much lower number of 175 T-DNA integrations into random T-DNA tagged lines, was less biased for a systematic analysis of T-DNA patterns in filamentous fungi. In addition, the GO annotation of targeted proteins was used to have a better insight into T-DNA integration mechanisms in *L. maculans*, which has not been done in fungi, except budding yeast, to date (Christie *et al.* 2009).

The ultimate goal of ATMT mutagenesis in plants or fungi is to reach saturation mutagenesis in order to eventually reach a functional annotation of the numerous unknown or hypothetical genes in the genomes. For example, in *L. maculans*, only 43% of the predicted proteins in the genome have strongly supported functional annotation, 45% are similar to hypothetical proteins for which no functional annotation is available, and 12% are predicted proteins with no annotation whatsoever (Rouxel *et al.* 2011; J. Grandaubert, unpublished data). In addition, in the case of phytopathogens, the initial objective of the T-DNA insertional mutagenesis strategy is the generation of mutants showing pathogenicity defects, as well as the discovery of novel genes and novel functions involved in pathogenesis. For these objectives, ATMT has to target mostly genic compartments of the genomes and show limited biases in targeted genes or genomic regions. The first advantage of ATMT for this objective is the common single-copy integration of the T-DNA in genomes, and mainly in fungal genomes, including that of *L. maculans* (Michielse *et al.* 2005; Blaise *et al.* 2007). The second point to be stressed in *L. maculans* is the high percentage of recovery of flanking sequences with matches in the fungal genome (around 80%) as was also observed for *M. oryzae* (Meng *et al.* 2007), whereas in plants, the frequencies usually amount to 60–65% [*e.g.* Thole *et al.* (2010)]. As shown for *M. oryzae*, or *Arabidopsis* and other model plant species, the T-DNA integration is shown here to be nonrandom. First T-DNA integration was much rarer than expected in TEs, as is generally the case in plants (Thole *et al.* 2010; Zhang *et al.* 2007), although this bias was not found when analyzing random transformants of *M. oryzae* (Meng *et al.* 2007). One possible explanation for this discrepancy would lie in the fact that all TEs in the genome of *L. maculans* are strongly degenerated and inactivated (Rouxel *et al.* 2011) and that, as discussed below, T-DNA integration favors transcriptionally active regions of the genome. Also, as is the case for *M. oryzae*, T-DNA tags were not recovered from other large arrays of repeats, such as the rDNA array, or from the mitochondrial genome (Meng *et al.* 2007), whereas the tagging of the rDNA array by T-DNA is overrepresented in some plant species, such as *B. distachyon* (Thole *et al.* 2010). Similarly to what was observed in *M. oryzae*, a marginal chromosomal bias showed some favored or disfavored chromosomes for T-DNA integration in *L. maculans*. As was noticed for *M. oryzae*, the biological significance of this fact remains obscure because no functional specificity was associated with these chromosomes. This bias may only be due to the limited number of tags analyzed in randomly tagged fungal isolates as it does not seem to occur in plants where the nonrandom integrations are observed within a chromosome rather than between chromosomes (Thole *et al.* 2010). More importantly, one main feature of T-DNA integration in the genome [*i.e.*, the favored targeting of 5′ 500-bp regions of genes assumed to be promoters] is a widely shared trait for plants and fungi (Alonso *et al.* 2003; Meng *et al.* 2007; Choi *et al.* 2007; Thole *et al.* 2010). By comparison with *M. oryzae*, the bias toward promoter regions was even more marked in the genome of *L. maculans*, consistent with the common recovery of pathogenic mutants for which the altered pathogenicity was due to T-DNA integration in promoters of genes (Elliott and Howlett 2006; Remy *et al.* 2008a,b, 2009). Lastly, the favored targeting of promoters is consistent with the presence of microhomology motifs (see below) involved in the homologous recombination with the T-DNA border.

When compiled, the observed T-DNA integration biases seem to share at least one common denominator: T-DNA integration takes place in transcriptionally active regions. In a cell, transcriptional activity should be considered the first step in the translation of genomic information into physiological state. Hence, starting from this postulate, it is logical to suppose that cellular activity affects T-DNA–favored insertion sites and that targeted genes should reflect, to a certain extent, the physiological state of the transformed cell. In this study, T-DNA insertion loci were recovered from *L. maculans* transformants obtained by ATMT for which germinating conidia (incubated for 48 hr) were used (Blaise *et al.* 2007). Conidial germination is commonly described as a three-step mechanism (D'enfert 1997; Osherov and May 2001): (i) activation, during which appropriate amounts of water and low-molecular-weight nutrients trigger conidial cell activation for germination; (ii) isotropic growth, during which the conidial cell undergoes morphological changes, uptakes water, and increases its physiological activity, which leads to an increase in size and mass; and (iii) polarized growth, during which a germ tube emerges from the conidial cell and develops, which requires *de novo* synthesis of wall materials. Germination is an asynchronic phenomenon that may differ from one conidia to another. ATMT is thus performed on conidial populations at four physiological stages: (i) ungerminated conidia, (ii) conidia at germination activation, (iii) conidia at isotropic growth, and (iv) conidia at polarized growth. The overrepresentation of “pigmentation,” “growth,” and “cell wall organization or biogenesis” functional categories in genes targeted by the T-DNA would be consistent with the hypothesis that targeted genes reflect the physiological state of the germinating conidia. First, even though the conidia of *L. maculans* are hyaline under the microscope, deposition of melanin and other pigments is generally associated with spore production in fungi in which they seem to function in the protection of microbes against environmental stress such as UV light and heat (Will *et al.* 1987; Brags *et al.* 2006; Rangel *et al.* 2006), consistent with the overrepresentation of the “pigmentation” category. Second, “growth” fits the isotropic growth step of conidial germination. Third, “cell wall organization or biogenesis” fits the polarized growth step of conidial germination.

T-DNA intranuclear targeting is assumed to result from a long evolution of *Agrobacterium* species' transfection mechanisms to fit host cellular machinery. Starting from this postulate, we analyzed this phenomenon mainly from the host point of view. Consequently, we considered T-DNA insertion biases not only as resulting from T-DNA characteristics but also largely depending on the following: (i) host genome characteristics (in *L. maculans* GC isochores/gene-rich *vs.* AT isochores/gene-poor compartments); (ii) gene expression at both transcriptional (machinery) and functional (cell physiological state) levels; and (iii) DNA features characterized by heterogeneity, unequal sensitivity to DNA damages, and organization in a gene-dependent fashion.

Locus biases, which are due to a chromatin-targeting process that guides T-DNA from its entry into the nucleus to its anchorage to host chromatin, were investigated. Previous studies have shown that T-DNA nuclear import is mediated by two bacterial virulence (Vir) proteins, VirD2 and VirE2, which directly associates with T-DNA to form the transport (T) complex [for review, see Tzfira *et al.* (2000) and Zupan *et al.* (2000)]. In addition to T-DNA encapsidation, Vir proteins act as interfaces with host machinery: VirD2 is phosphorylated *in vivo* by CAK2M, a cyclin-dependent kinase-activating kinase, and is tightly associated with TATA box-binding protein (TBP) (Bakó *et al.* 2003), and VirE2 binds VIP1 (VirE2-interacting protein 1), a bZIP transcription factor capable of binding core histones (Li *et al.* 2005; Loyter *et al.* 2005). VIP1, CAK2M, and TBP profile T-DNA insertion loci distribution within the host genome, and their proper functions and properties generate locus biases: CAK2M phosphorylates the C-terminal domain of the RNA polymerase II (RNA Pol II) largest subunit (Bakó *et al.* 2003), which serves as a TBP-binding platform (Yuryev *et al.* 1996); TBP binds the TATA box core promoter element, whose recognition nucleates the assembly of transcription preinitiation complex [for review, see Smale and Kadonaga (2003)]; and VIP1 precisely binds a DNA hexamer motif found in the promoters of various stress-responsive genes and plays a role in immunity signaling by stimulating stress-dependent gene expression, at least in plants (Djamei *et al.* 2007; Pitzschke *et al.* 2009). However, because VIP1 shows no significant homology to known animal or fungal proteins, it could be plant specific. Nevertheless, because T-complex anchorage to host chromatin seems to be a key step for further T-DNA integration, it is consistent to consider the existence of animal and fungal VIP1-like proteins interacting with both T-complex and host chromatin. The T-DNA insertion pattern in *L. maculans* corroborates this tight relation between T-DNA and gene transcription machinery, because T-DNA insertions were predominant in the following: (i) GC isochores, which are gene-rich islands frequently targeted by gene transcription machinery and therefore more likely to be in a relaxed, opened state, rather than AT isochores, which are TE-rich, gene-poor regions assimilated to heterochromatin, therefore condensed and closed (Rouxel *et al.* 2011); (ii) gene-regulatory regions, which border zones between a histone-containing region capable of anchoring the T-complex and a histone-less region that is the gene-expressing DNA; and (iii) promoter region, in which additional binding opportunities (CAK2M, TBP) increase the probability of T-complex anchorage and strengthen it.

T-complex anchorage to host chromatin is not synonymous with T-DNA insertion. For the latter to occur, DNA damage is mandatory, because the T-DNA integration process abuses HR and NHR pathways, two host-DNA double-strand break (DSB) repair machineries [for review, see Tzfira *et al.* (2004) and Citovsky *et al.* (2007)]. Consequently, as additional factors that affect occurrence of T-DNA integration events, we must consider both DSBs hotspots and DSB repair efficiency. In eukaryotic cells, DSBs are common events resulting from both environmental and endogenous factors. DSBs are also created by converting single-strand lesions (Natarajan 1993) and retrotransposon activity (Gasior *et al.* 2006), and they occur preferentially in opened chromatin (Berchowitz *et al.* 2009) and transcriptionally active promoters, telomeres, and centromeres (Wu and Lichten 1994; Baudat and Nicolas 1997; Blitzblau *et al.* 2007; Buhler *et al.* 2007). However, not all occurring DSBs are repaired with the same efficiency. In fact, telomeric regions and packed heterochromatin are deficient in repair of DSBs (Ricchetti *et al.* 2003). Together, these studies highlight biases of DSB occurrence and repair that correlate with T-DNA mapping biases in *L. maculans*. Actually, T-DNA integration favored gene-rich GC isochores and not AT isochores that exhibit heterochromatin characteristics in which chromatin is packed and DSB repair is likely to be deficient, and T-DNA integration events overmapped to gene promoter regions where DSBs are assumed to occur frequently.

To corroborate mapping biases in T-DNA integration events, we analyzed T-DNA LB, T-DNA preinsertion and insertion site sequences, and T-DNA–targeted genes for particular compositional, structural, and functional signatures, and we showed that T-DNA LB shares microhomologies with preinsertion sites, suggesting that T-DNA integration may occur at least by HR in *L. maculans*. The same was observed by Meng *et al.* (2007) and by Choi *et al.* (2007) in the *M. oryzae* genome, but the authors did not reach conclusive evidence regarding the targeted motifs. Hence, T-DNA LB sequence affinity with host DNA may affect T-DNA integration event distribution. Our results highlighted that T-DNA LB harbored microhomologies with CAAT box, Inr, and TATA box of eukaryotic promoters. Also, TATA-containing micromology motifs were frequently shared between T-DNA LB and its target sequence. These observations correlate with frequent mapping of T-DNA insertions to gene promoter regions, in the sense that sequence affinity is mandatory for DNA end joining by MMEJ [for review, see McVey and Lee (2008)].

DNA asymmetry was observed in both prokaryotic and eukaryotic genomes. It is a consequence of many mechanisms, among which gene expression is one of the better studied, and in which DNA asymmetry is seen as signatures indicating functional signals and DNA modifications (Touchon and Rocha 2008). DNA asymmetry is revealed by the CG skew and AT skew. Previous studies noticed that the CG skew is stronger than the AT skew, at least in eubacteria (Francino and Ochman 1997; Frank and Lobry 1999; Tillier and Collins 2000) and that skew curves are associated with replication origin (Lobry, 1996a,b; Blattner *et al.* 1997; Kunst *et al.* 1997; Grigoriev 1998) and transcription-coupled and splicing-coupled mutations (Touchon *et al.* 2004). In particular, CG skew peak is associated with gene expression level, at least in plants (Alexandrov *et al.* 2006), and transcription initiation starts in eukaryotes, including fungi (Tatarinova *et al.* 2003; Fujimori *et al.* 2005; Alexandrov *et al.* 2006). Altogether, these studies highlight a correlation between DNA asymmetry and cellular activity–driven DNA manipulations and modifications in general (replication, gene expression, mutations) and a tight association between an increased CG skew and gene transcription in particular. T-DNA targeted to CG asymmetric DNA is thus consistent with frequent insertions in transcriptionally active regions and gene promoter sequences.

## CONCLUSIONS

Using a fungal genome showing contrasted genomic landscapes, our data substantiate the advantages of ATMT to reach a functional annotation of genes, but they cast doubts on whether this strategy will be able to target species-specific genes involved in pathogenicity that reside in specific AT-rich compartments of the fungal genome. The main particularities substantiating these points are (i) the common single-copy integration of the T-DNA; (ii) the high frequency of integration within protein-coding genes (even if the introns are favored targets), amounting to one third of the integration events in *L. maculans*; and (iii) the common occurrence in promoters favoring the access to genes whose complete inactivation would be too detrimental for the fungus. In contrast, under the hypothesis that genes specifically involved in plant pathogenicity are hosted in specific compartments of the genome, we notice that T-DNA targeting to AT isochores is very low compared with the percentage of these landscapes in the genome, but it is nevertheless consistent with the amount of genes hosted in AT isochores. More importantly, our work suggests the importance of the physiology of the fungus at the time of ATMT and the favored targeting of transcriptionally active regions of the genome. With most of the genes involved in pathogenicity, such as those encoding effector proteins, repressed during the vegetative growth of the fungus and overexpressed at the onset of plant infection (Rouxel *et al.* 2011), these are unlikely to be targeted by the T-DNA.
